# Mesenchymal Stromal Cell Therapy in Novel Porcine Model of Diffuse Liver Damage Induced by Repeated Biliary Obstruction

**DOI:** 10.3390/ijms22094304

**Published:** 2021-04-21

**Authors:** Lucie Vištejnová, Václav Liška, Arvind Kumar, Jana Křečková, Ondřej Vyčítal, Jan Brůha, Jan Beneš, Yaroslav Kolinko, Tereza Blassová, Zbyněk Tonar, Michaela Brychtová, Marie Karlíková, Jaroslav Racek, Hynek Mírka, Petr Hošek, Daniel Lysák, Milena Králíčková

**Affiliations:** 1 Biomedical Center, Faculty of Medicine in Pilsen, Charles University, Alej Svobody 76, 323 00 Pilsen, Czech Republic; Lucie.Vistejnova@lfp.cuni.cz (L.V.); arvind.kumar896@gmail.com (A.K.); jaja.houbina@gmail.com (J.K.); vycitalo@fnplzen.cz (O.V.); bruhaj@fnplzen.cz (J.B.); benesj@fnplzen.cz (J.B.); Yaroslav.Kolinko@lfp.cuni.cz (Y.K.); Tereza.Blassova@lfp.cuni.cz (T.B.); Zbynek.Tonar@lfp.cuni.cz (Z.T.); misstery007@gmail.com (M.B.); karlikovam@fnplzen.cz (M.K.); mirka@fnplzen.cz (H.M.); petr.hosek@lfp.cuni.cz (P.H.); lysak@fnplzen.cz (D.L.); milena.kralickova@lfp.cuni.cz (M.K.); 2 Department of Histology and Embryology, Faculty of Medicine in Pilsen, Charles University, Karlovarska 48, 301 00 Pilsen, Czech Republic; 3 Department of Surgery, Medical School and Teaching Hospital in Pilsen, Charles University, Alej Svobody 80, 304 60 Pilsen, Czech Republic; 4 Department of Anesthesiology and Intensive Care, Medical School and Teaching Hospital in Pilsen, Charles University, Alej Svobody 80, 304 60 Pilsen, Czech Republic; 5 Department of Clinical Biochemistry and Hematology, Medical School and Teaching Hospital in Pilsen, Charles University, Alej Svobody 80, 304 60 Pilsen, Czech Republic; racek@fnplzen.cz; 6 Department of Radiology, Medical School and Teaching Hospital in Pilsen, Charles University, Alej Svobody 80, 304 60 Pilsen, Czech Republic

**Keywords:** secondary biliary cirrhosis, hepatectomy, pig model, mesenchymal stromal cell, quantitative histology

## Abstract

In liver surgery, biliary obstruction can lead to secondary biliary cirrhosis, a life-threatening disease with liver transplantation as the only curative treatment option. Mesenchymal stromal cells (MSC) have been shown to improve liver function in both acute and chronic liver disease models. This study evaluated the effect of allogenic MSC transplantation in a large animal model of repeated biliary obstruction followed by partial hepatectomy. MSC transplantation supported the growth of regenerated liver tissue after 14 days (MSC group, *n* = 10: from 1087 ± 108 (0 h) to 1243 ± 92 mL (14 days); control group, *n* = 11: from 1080 ± 95 (0 h) to 1100 ± 105 mL (14 days), *p* = 0.016), with a lower volume fraction of hepatocytes in regenerated liver tissue compared to resected liver tissue (59.5 ± 10.2% vs. 70.2 ± 5.6%, *p* < 0.05). Volume fraction of connective tissue, blood vessels and bile vessels in regenerated liver tissue, serum levels of liver enzymes (AST, ALT, ALP and GGT) and liver metabolites (albumin, bilirubin, urea and creatinine), as well as plasma levels of IL-6, IL-8, TNF-α and TGF-β, were not affected by MSC transplantation. In our novel, large animal (pig) model of repeated biliary obstruction followed by partial hepatectomy, MSC transplantation promoted growth of liver tissue without any effect on liver function. This study underscores the importance of translating results between small and large animal models as well as the careful translation of results from animal model into human medicine.

## 1. Introduction

Repeated or chronic biliary obstruction is a common result of choledocholithiasis, cholangitis or postoperative complications of cholecystectomy. Clinically, biliary obstruction can manifest as abdominal pain, fever, itching, dark urine and jaundice, but it can also give rise to more severe complications such as portal hypertension (PH), acute cholangitis, vitamin deficiencies and bacterial infections [1]. However, the most severe consequence of chronic cholestasis is secondary biliary cirrhosis (SBC), a life-threatening disease with liver transplantation as the only curative treatment option [2,3,4,5,6]. In cases of benign obstruction, cirrhosis can develop within 14–62 months, while malignant cases can quickly develop into SBC in as few as 9 months [1,4,7]. 

Common treatments for cholestasis often begin with attempts at drainage, stent placement or, if choledocholithiasis is suspected, then endoscopic retrograde cholangiopancreatography and stone removal. In more severe cases of liver damage, the initial course of treatment usually involves management of symptoms. For example, ursodeoxycholic acid is often prescribed to improve liver function, while other drugs can be used to absorb circulating bile acids [8]. However, in cases of SBC, the only curative treatment that exists to control disease spreading and liver failure is liver transplantation [2,4,5]. Liver transplantation has been associated with good long-term outcomes for patients with SBC, increasing 5-year survival rates to 69–75% [4,9,10]. Nevertheless, liver transplantation is only recommended in severe cases that exhibit functional changes in the liver. Furthermore, on top of the existing limitations surrounding liver transplantation, additional long-term complications associated with biliary obstruction can also hinder surgical efficacy. By the time patients are considered for liver transplantation, they have likely already undergone several, often failed, operations, which can serve as an obstacle for future surgical repair [11,12]. Moreover, the high risk of PH in cirrhotic patients can lead to increased secondary bleeding during surgery, and the presence of PH can even increase 60-day postoperative mortality rates for patients undergoing any surgery for bile duct strictures from 2% to 23% [2,3,13,14].

Given the severity of complications associated with liver transplantation for patients with chronic cholestasis that can develop into SBC, it is necessary to develop an alternative method that is still a curative, as opposed to symptomatic, treatment. One such option may arise from referring to treatment methods for other forms of cirrhosis. Specifically, the use of mesenchymal stromal cells (MSC) to cure liver cirrhosis has had positive results in both in vivo research and clinical trials. In mice, bone marrow-derived MSC were used to successfully ameliorate carbon tetrachloride (CCl4)-induced liver fibrosis by expressing a VEGF receptor that stimulated hepatic regeneration and collagen deposition [15]. These results were expanded by Li et al., who found that MSC can promote liver regeneration after portal vein embolization in cirrhotic rats [16]. Additional aspects about the treatment mechanism were elucidated by Higashiyama et al., who found that MSC express matrix metalloproteinase-9 with antifibrotic effects, and Oyagi et al., who discovered that culturing MSC with hepatocyte growth factor can improve their anti-fibrotic effects [17,18]. Similar results were found in human clinical trials by Jang et al. and Amin et al., which showed that autologous bone marrow-derived MSC can treat alcoholic cirrhosis as well as post-hepatitis C virus cirrhosis [19,20].

As such, although liver transplantation can be an effective treatment for SBC, the potential complications associated with surgery in severe cases inhibit its use. Thus, it is necessary to find a different method of treatment that still holds the same curative properties. MSC have the potential to treat biliary cirrhosis by both non-parenchymal cell response modulation as well as supporting and stimulating the regeneration of nascent hepatocytes [15,16]. In this study, the efficacy of allogenic bone-marrow derived MSC on liver regeneration was analyzed in a novel porcine model of repeated biliary obstruction and partial liver resection.

## 2. Results

### 2.1. Evaluation of MSC Phenotype and Differentiation Ability

**Figure 1:**
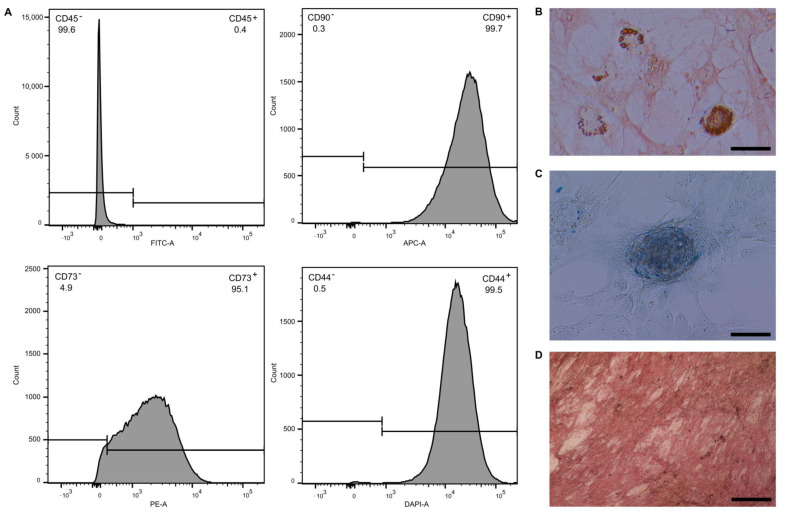
Evaluation of mesenchymal stromal cell (MSC) phenotype by flow cytometry and differentiation potential examination. Representative histograms show that MSC- were negative in CD45 and positive in CD90, CD73 and CD44 (**A**). Differentiation potential of transplanted MSC was evaluated by their differentiation into: adipo-lineage (**B**); chondro-lineage (**C**); and osteo-lineage (**D**). The scale bar in the pictures represents 100 μm.

Stem cell phenotype of transplanted MSC was evaluated by flow cytometry. MSC were transplanted after the 3rd passage to ensure both minimal loss of stem cell characteristics and minimal difference between MSC groups. Transplanted MSC were positive in CD90 (99.7%), CD73 (95.1%) and CD44 (99.4%) and negative in CD45 (0.40%) (Figure 1A). Differentiation ability of transplanted MSC was evaluated by differentiation into adipo-, chondro- and osteo-lineages. After 21 days of differentiation, MSC were able to accumulate lipid droplets, detected by oil red staining (Figure 1B) as a marker of adipo-lineage differentiation; produce glycosaminoglycans, detected by alcian blue staining (Figure 1C) as a marker of chondro-lineage differentiation; and deposit calcium cations, detected by alizarin red staining (Figure 1D) as a marker of osteo-lineage differentiation.

### 2.2. Portal Vein Transplantation of MSC Stimulated Liver Regeneration in a Porcine Model of Biliary Obstruction

**Figure 2:**
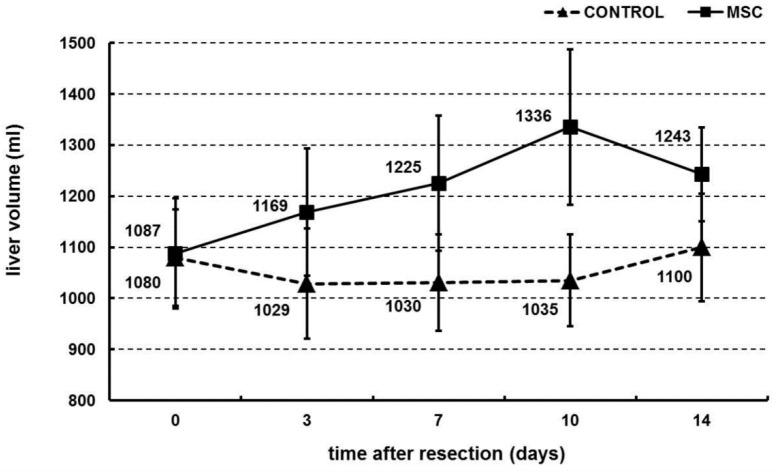
Volumetry of regenerated liver. Left lobe of liver was resected and volume (mL) was measured ultrasonographically 0, 3, 7, 10 and 14 days after resection. Mesenchymal stromal cells (MSC, 10^6^ cells/kg; *n* = 10) or physiological solution (CONTROL, *n* = 11) were transplanted by portal vein into the liver immediately after resection. Mean ± standard deviation is shown. The difference in liver volume trends over time between the MSC group and control group was significant (Mann–Whitney U test of cross-ranked values, *p* < 0.05).

Regenerated liver volume was measured 3, 7, 10 and 14 days after left lobe liver resection and MSC portal vein transplantation. At all time points, the MSC group exhibited a higher liver volume than the control group (Figure 2). Liver volume increased over time in the MSC group, from 1087 ± 108 mL (0 h) to 1243 ± 92 mL (14 days). On the other hand, liver volume remained constant in the control group, starting at 1080 ± 95 mL (0 h) and ending at 1100 ± 105 mL (14 days) (Mann–Whitney U test of cross-ranked values, *p* = 0.016).

### 2.3. Portal Vein Transplantation of MSC Slightly Modulated Morphometric Parameters of the Regenerated Liver in a Porcine Model of Biliary Obstruction

**Figure 3:**
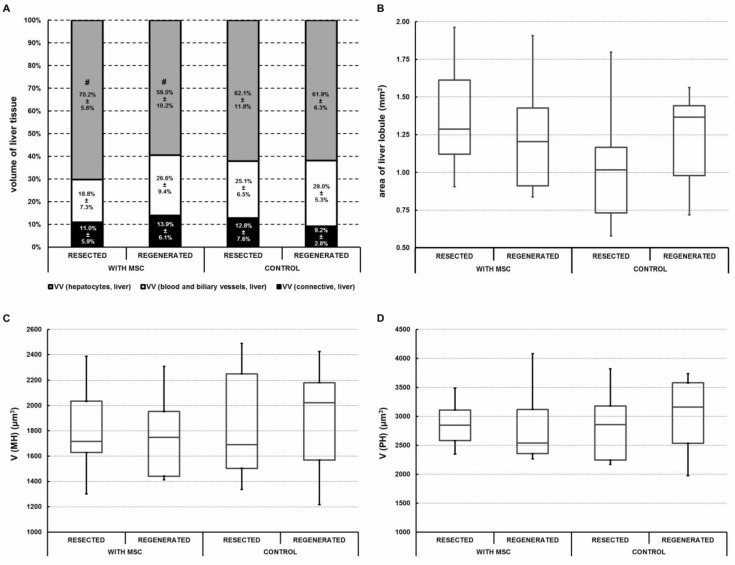
Quantitative morphometric analysis of liver before (resected, 0 day) and after regeneration (regenerated, 14 days) in the mesenchymal stromal cells (MSC) and control groups. (**A**) Significant differences were found between the volume fractions of hepatocytes V_V_ (hepatocytes, liver) before and after regeneration in the MSC group (#, Wilcoxon matched pairs test *p* < 0.05). Volume fractions of connective tissue V_V_ (connective, liver) and blood and biliary vessels V_V_ (blood and biliary vessels, liver) exhibited no significant difference between the groups. (**B**) No significant differences were found between the groups when comparing the mean areas of liver lobules A(lobule). (**C**,**D**) No significant differences were found when comparing the mean volume of mononuclear hepatocytes V(MH) or the mean volume of polynuclear hepatocytes V(PH). Grey color = V_V_ (hepatocytes, liver); black color = V_V_ (connective, liver); white color = V_V_ (blood and biliary vessels, liver).

Quantitative morphometric analysis was performed on resected and regenerated liver samples and compared in both the MSC and control groups. No large areas with steatosis were observed in any group. No differences in histological morphometric parameters were found when comparing the samples from resected (0 day) and regenerated (14 days) livers in both groups (Figure 3A). The MSC group had a smaller VV (hepatocytes, liver) in regenerated samples (59.5 ± 10.1%) than in samples before regeneration (70.2 ± 5.5%) (Wilcoxon matched pairs test *p* = 0.013) (Figure 3A. with MSC). On the other hand, percentage division remained unchanged before and after regeneration in the control group (Figure 3A. control). No statistical significance was found when comparing the differences between resected and regenerated liver samples with regards to the area of liver lobules (Figure 3B), the volume of mononuclear hepatocytes (Figure 3C) or the volume of polynuclear hepatocytes (Figure 3D) between the MSC and control group. The correlations between morphometric parameters are presented separately for both groups in Table 1. In the MSC group, the volume fraction of connective tissue within the regenerated liver was negatively correlated with the mean volume of hepatocytes (R = −0.78 in mononuclear hepatocytes and R = −0.70 in polynuclear hepatocytes), i.e., regions with more connective tissue contained smaller hepatocytes. This correlation was absent in the control group. In the MSC group, the volume fraction of both mononuclear and polynuclear hepatocytes within the regenerated liver was also strongly correlated with the volume fraction of hepatocytes (R = 0.69), i.e., regions containing regenerated hepatocytes were populated by mainly mononuclear hepatocytes. This correlation was much weaker (R = 0.23) in the control group. 

### 2.4. Portal Vein Transplantation of MSC Did Not Affect Biochemical Parameters in a Porcine Model of Biliary Obstruction

**Figure 4:**
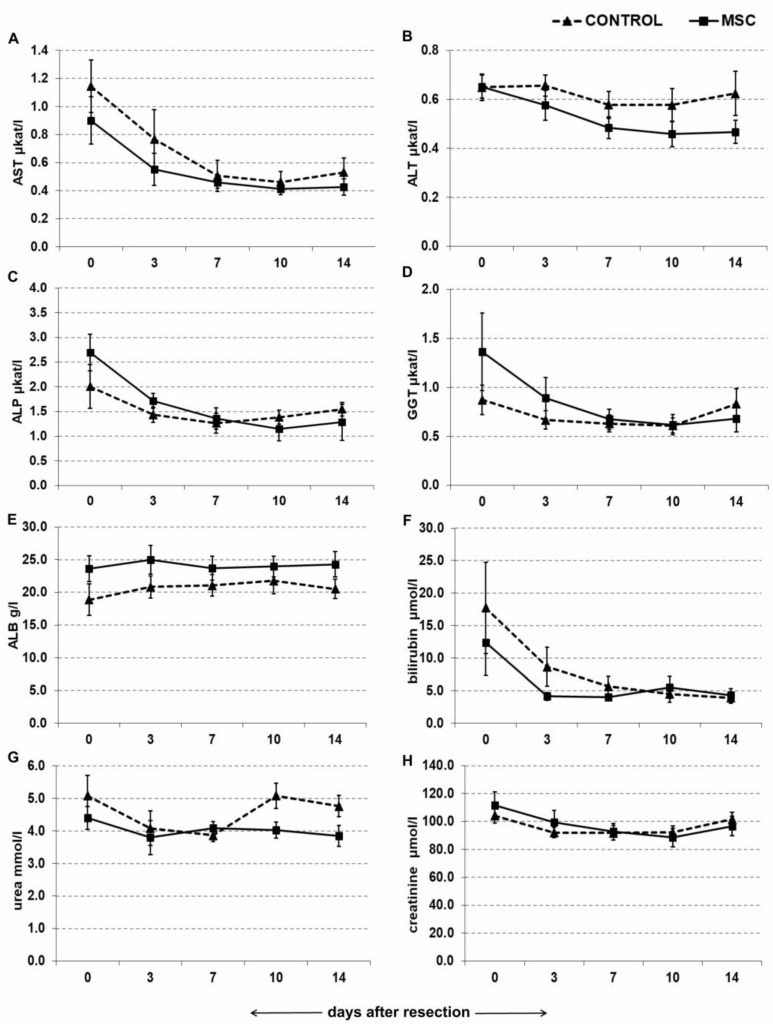
Quantification of liver enzymes and metabolites after liver resection. Mesenchymal stromal cells (MSC, 10^6^ cells/kg; *n* = 10) or physiological solution (CONTROL, *n* = 11) were transplanted by portal vein into liver immediately after resection. Concentration of liver enzymes and metabolites was measured by established biochemical methods 0, 3, 7, 10 and 14 days after resection. Mean ± standard deviation is shown. The difference in trends of concentration change over time between the MSC group and control group was not significant for any liver enzyme or metabolite (Mann–Whitney U test of cross-ranked values, *p* < 0.05). (**A**) AST, aspartate transaminase; (**B**) ALT, alanine transaminase; (**C**) ALP, alkaline phosphatase; (**D**) GGT, γ-glutamyl transferase; (**E**) ALB, albumin; (**F**) bilirubin; (**G**) urea; (**H**) creatinine.

Quantification of key biochemical parameters was performed to monitor the effect of MSC transplantation via portal vein. Observed parameters included aspartate transaminase (AST), alanine transaminase (ALT), alkaline phosphatase (ALP), γ-glutamyl transferase (GGT), albumin (ALB), bilirubin, urea and creatinine. After resection, AST, ALP and GGT continued to decrease in both the MSC and control groups, but a significant difference was not found between the groups (Figure 4A,C,D). ALT decreased in the MSC group and remained constant in the control group, but the difference between the two groups was not found to be statistically significant (Figure 4B). ALB, urea and creatinine remained constant overall (Figure 4E,G,H), except on Days 3 and 7 when urea levels decreased slightly in both groups (Figure 4G). Finally, bilirubin levels decreased in both groups, but there was no significant difference between the two (Figure 4F).

### 2.5. Portal Vein Transplantation of MSC Did Not Modulate Pro-Inflammatory Cytokines and TGF-β in a Porcine Model of Biliary Obstruction

**Figure 5:**
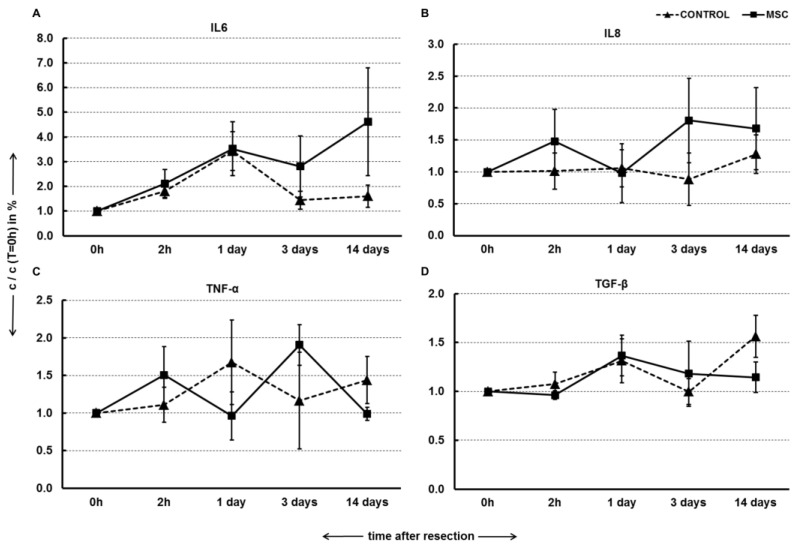
Quantification of pro-inflammatory cytokines and TGF-β. Left lobe of liver was resected and mesenchymal stromal cells (MSC, 10^6^ cells/kg; *n* = 10) or physiological solution (CONTROL, *n* = 11) were transplanted by portal vein into liver immediately after resection. Concentration of IL6 (**A**), IL8 (**B**), TNF-α (**C**) and TGF-β (**D**) was measured by ELISA 0 h, 2 h, 1 day, 3 days and 14 days after resection. Concentrations at particular times are normalized to the concentration before MSC transplantation (c(T)/c(T = 0 h)). Mean ± standard deviation is shown. The difference in trends of concentration change over time between the MSC group and control group was not significant for any analyte (Mann–Whitney U test of cross-ranked values, *p* < 0.05).

Quantification of pro-inflammatory cytokines and anti-inflammatory TGF-β was performed to monitor the immune-modulation potential of MSC transplantation via portal vein. Observed cytokines included IL6, IL8, TNF-α and TGF-β. IL6 increased at both 2 h and 1 day after resection in a similar manner for both groups. By Day 14 after resection, the MSC group exhibited a much greater increase in IL6 levels compared with the control group. However, these differences did not show any statistical significance (Figure 5A). IL8 levels remained constant after resection in the control group, while a net increase was observed in MSC group, but these results were not significant (Figure 5B). TNF-α levels fluctuated throughout the 14 days in both groups (Figure 5C). Finally, 1 day after resection, TGF-β levels increased in both groups but remained constant thereafter in the MSC group and continued to increase in the control group. However, the differences between the two groups were not statistically significant (Figure 5D).

## 3. Discussion

The present study employed a novel porcine model of repeated biliary obstruction followed by left lobe resection in order to simulate clinical appearance and intervention of chronic cholestasis that can lead to SBC. After resection, MSC were transplanted via the portal vein, and their effects were estimated by volumetric, histological, biochemical and immunological analysis. The results show that portal vein transplantation of MSC stimulated growth of liver volume during regeneration (Figure 2) and slightly modulated morphometric parameters of regenerated liver parenchyma (Figure 3 and Table 1) over a 14-day period after liver resection. However, MSC transplantation did not affect liver biochemical parameters (Figure 4) or pro-inflammatory cytokines and TGF-β (Figure 5), which play a role in liver function and liver regeneration, respectively.

This study was the first of its kind to utilize a porcine model of intermittent biliary obstruction and benefits from its novelty on two fronts. First, even among studies that tested MSC as treatment for liver cirrhosis, none were found to use a large animal model, instead using rodents to replicate the disease [15,16]. Using a porcine model of repeated biliary obstruction offers greater insight and translatability than small animal models for human disease. Second, due to commonly inducing continual biliary obstruction in rats, prior models have lacked true clinical relevance [21,22,23]. Even the few studies found that used porcine models of biliary obstruction studied acute cholangitis and relied upon short-term (7 or 15 day) bile duct ligation at a single timepoint, similar to the previous rat models [24,25,26]. 

**Figure 6:**
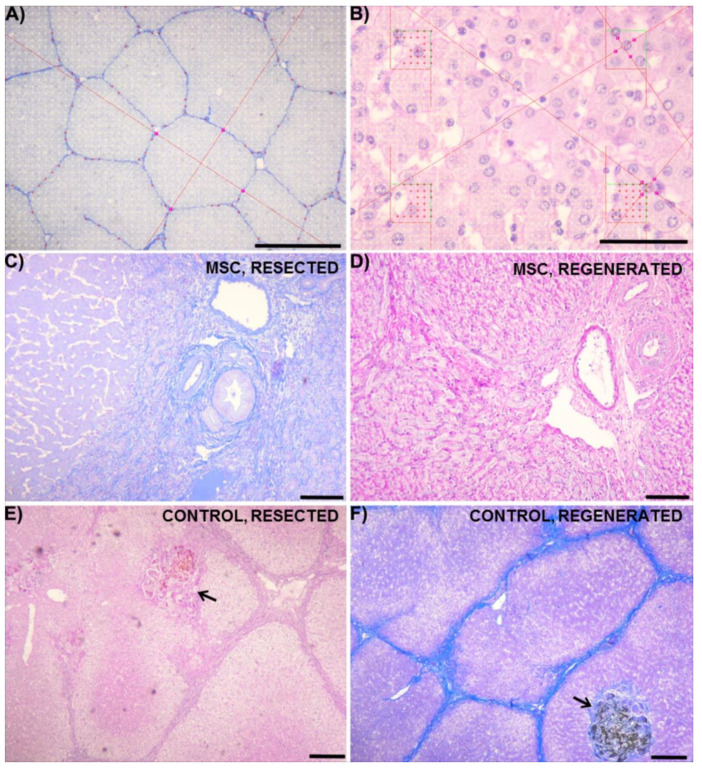
Quantitative histological analysis and examples of liver morphology. (**A**) The volume fraction of connective tissue within the liver was estimated using a point grid (yellow). Cross-sectional area of hepatic lobules was estimated using a two-dimensional nucleator probe (red). (**B**) In hepatocytes selected using the counting frames, the volume fraction of hepatocytes within the liver was quantified using the point grid (red marks). The mean volume of individual hepatocytes was estimated using the nucleator probe (red lines with intercepts on the edge of hepatocytes). The areas of portal triads in MSC (mesenchymal stromal cells) groups in resected (**C**) and regenerated (**D**) liver samples are shown. Overall morphological pictures of liver structure are shown for control groups in resected (**E**) and regenerated (**F**) liver samples without apparent differences in compared areas. Isolated areas of bile obstruction (arrows) are shown in (**E**,**F**). Alcian blue and nuclear red staining (**A**,**C**,**F**) and PAS stain (**B**,**D**,**E**) was used. Scale bars correspond to: 500 μm (**A**); 50 μm (**B**); 100 μm (**C**,**D**); and 200 μm (**E**,**F**).

While some studies have claimed benefit from successfully inducing SBC, they tend to lack translatability to humans, as very few patients suffer from such prolonged obstruction that develops into SBC. Instead, SBC is most commonly developed after repeated biliary obstruction [2,3,4,5,6], which was achieved in this study by the successful use of Fogarty’s catheter to generate a cycle of obstruction each week. Histological analysis also demonstrated isolated areas of biliary obstruction visible in liver parenchyma (Figure 6E,F), confirming morphological changes in the liver due to the repeated obstruction. Thus, this model better replicates clinically relevant cases of chronic human cholestasis and biliary obstruction, including those that eventually develop into SBC.

In its application, according to volumetric analysis, the model used in this study showed that MSC may serve as a viable treatment strategy to support liver regeneration. Initial results indicated that MSC significantly aided in increasing liver volume after partial resection in pigs experiencing repeated biliary obstruction. These results are in line with previous studies that have shown that MSC are capable of stimulating liver regeneration by increasing liver volume and/or weight. Adas et al. showed that MSC transplantation significantly increased liver volume, both with and without additional transfection of VEGF, after 70% resection in rats [27]. With regards to cirrhosis, Li et al. showed that autologous MSC transplantation successfully resulted in a higher regeneration response of future liver remnant in CCL4-induced cirrhotic rats that experience portal vein embolization [16]. Finally, in a small-scale clinical study, Mohamadnejad et al. showed that MSC successfully increased liver volume in patients with decompensated liver cirrhosis over a six-month timespan [28].

However, MSC transplantation did not improve liver function based on plasma levels of liver enzymes. Similar results were obtained in studies utilizing severe liver failure models in which unchanged levels of transaminases and albumin were also detected in both MSC-treated and control groups [29,30]. Plasma levels of pro-inflammatory cytokines IL-6, IL-8 and TNF-α and anti-inflammatory TGF-β also remained unaffected by MSC transplantation in our study, highlighting the questionable ability of MSC to modulate immune response upon injection into liver, where the cooperation of immune cells and MSC is still a matter for debate. Even though MSC has the potential to be delivered to the liver [31], a possible explanation for this result is that MSC can be phagocytosed and eliminated by activated monocytes [32]. In the liver, resident Kupffer cells can interact with injected MSC and switch into anti-inflammatory and anti-fibrotic stages, but this has only been shown in vitro or in rodent models [33]. The anti- or pro-fibrotic behavior of MSC is also contested in current research. Although many studies showed that α-SMA expression, the volume of fibrotic tissue or hepatic stellate cell activity is ameliorated upon MSC application [34,35], an increasing number of studies has found contrary results [36,37].

The distribution of volume, nuclearity, and density within a healthy porcine liver was recently mapped [38]. Despite extensive sampling, no differences were identified between porcine liver lobes. Therefore, we believe that the differences reported in the present study were caused by the experiment rather than by random biological variability between the various parts of the porcine liver. The mean volume of all hepatocytes in rat models of cadmium-induced hepatic damage [39] was greater than that of the porcine mononuclear hepatocytes in our study, but similar to that of polynuclear hepatocytes. The volume of hepatocytes in our study also matches values obtained in an experiment testing the effects of cyclosporin A in rat livers [40].

This study yielded interesting relationships between morphometric parameters in pigs who received MSC treatment. When compared with the control group, the MSC group showed different correlation patterns between the connective tissue fraction and volume of both mononuclear and polynuclear hepatocytes. The reduced volume fraction of hepatocytes within the liver on Day 14 after MSC transplantation was accompanied by insignificant increases in the volume fractions of the complementary fractions, i.e., the connective tissue fraction and the fraction of blood and biliary vessels. A possible explanation of this finding is that MSC transplantation can stimulate proliferation of connective tissue [36], thus reducing the space for parenchymal hepatocytes.

Secondly, pigs in the MSC group exhibited a strong correlation between VV (hepatocytes, liver) and the fractions of mononuclear and polynuclear hepatocytes on Day 14 after resection. Specifically, the results indicate that regions containing more regenerated hepatocytes were mainly populated by mononuclear hepatocytes. A similar, but weaker, correlation was found in the control group as well. As an increase in polynuclear hepatocytes may be interpreted as a late consequence of oxidative stress [41], these results may indicate an inclination towards a better microenvironment for hepatocyte regeneration with an increased fraction of mononuclear hepatocytes in these regions. However, further experiments would be necessary to test these relationships and to interpret the biological roles of mononuclear and polynuclear hepatocytes during liver regeneration.

These two morphological correlations present in the MSC group may also explain the differences between the volumetric and biochemical effects of MSC transplantation. While the increase in connective tissue and vessels in the liver and decrease in number of hepatocytes may have resulted in increased liver volume overall, the biochemical parameters that reflect hepatocyte function would not have changed. This may also suggest that MSC function through a paracrine effect on hepatic stellate cells, endothelial cells and cholangiocytes, a result that has been supported in previous studies as well [42,43].

## 4. Materials and Methods

### 4.1. MSC Isolation and Culture

Porcine mesenchymal stromal cells (MSC) were isolated from healthy pigs (*Sus Scrofa*). Bone marrow from the tibia or femur bones was aspirated into 50 mL tubes (Techno Plastic Products, TPP, Trasadingen, Switzerland) containing heparin (B Braun, Melsungen, Germany) by puncture with a sterile needle. MSC were isolated from bone marrow by gradient centrifugation (440× *g*, 30 min) on Ficoll-Paque Plus (GE Healthcare, Chicago, IL, USA). The layer of mononucleated cells was washed with phosphate buffer saline and plated on a 75 cm^2^ culture flask (TPP) in culture media containing α-MEM cell culture media (Thermo Fisher Scientific, Waltham, MA, USA) supplemented by 10% fetal bovine serum (Thermo Fisher Scientific, Waltham, MA, USA), 1 mM L-glutamine (Biochrom, Cambridge, United Kingdom), 6.0 mg/mL penicillin, 10 mg/mL streptomycin (Biosera, Nuaille, France) and 0.25 mg/mL gentamicin (Biosera, Nuaille, France). Culture media was changed every second day. After 10 days, MSC were harvested by EDTA/Trypsin 1x (Biosera, Nuaille, France) and separated between three 75 cm^2^ culture flasks (TPP). Culture media was again changed every second day, and, after 10 days, MSC were harvested by EDTA/Trypsin (Biosera, Nuaille, France) and cryopreserved in liquid nitrogen (1 × 10^6^ cells/cryotube). Four weeks before transplantation, MSC were thawed, plated on 150 cm^2^ flasks (TPP) in 20 mL of the culture media described above and cultured for 4 weeks to get about 50 million cells with one passage cycle, maintaining the stem cell properties of MSC. On the day of transplantation, MSC were harvested as described above, counted, re-suspended in 100 mL of physiological solution (B Braun, Melsungen, Germany) (37 °C, 10^6^/kg of pig weight) per pig and immediately transplanted. Stem cell phenotype of transplanted MSC was identified by flow cytometry and differentiation ability.

### 4.2. Identification of MSC by Flow Cytometry

Before transplantation, stem cell phenotype of MSC was evaluated by flow cytometric detection of CD90, CD73 and CD44. First, 500,000 MSC were washed with PBS and stained with 5 µL of APC-CD90 (Biolegend, San Diego, CA, USA), PE-CD73 (Biolegend, San Diego, CA, USA), BV421-CD44 (Biolegend, San Diego, CA, USA) and FITC-CD45 (Bio-Rad, Hercules, CA, USA) for 15 min in the dark at room temperature. After staining, MSC were washed once, resuspended in 300 µL of PBS and analyzed by BD FACS Aria Fusion (Becton Dickinson, Franklin Lakes, NJ, USA) and BD FACS Diva 8.0.1 software (Becton Dickinson, Franklin Lakes, NJ, USA).

### 4.3. Adipogenic, Osteogenic and Chondrogenic Differentiation Ability of MSC

Differentiation ability of transplanted MSC was evaluated by their differentiation into adipo-, osteo- and chondro-lineage. MSC were seeded onto 12-well cultivation dishes (TPP) with a seeding density of 3.8 × 104 cells/well for adipogenic and chondrogenic differentiation and 1.9 × 104 cells/well for osteogenic differentiation in culture media. After a 24-h attachment period, the media was discarded and replaced with 3 mL of specific differentiation media: StemPro^®^ Adipogenesis Differentiation Kit (Thermo Fisher Scientific, Waltham, MA, USA) for adipogenic differentiation, StemPro^®^ Chondrogenesis Differentiation Kit (Thermo Fisher Scientific, Waltham, MA, USA) for chondrogenic differentiation and StemPro^®^ Osteogenesis Differentiation Kit (Thermo Fisher Scientific, Waltham, MA, USA) for osteogenic differentiation. After the differentiation period of 21 days, oil red O (Sigma Aldrich, St. Louis, MO, USA) staining for lipid droplet visualization in adipogenesis, alcian blue (Sigma Aldrich, St. Louis, MO, USA) staining for glycoprotein visualization in chondrogenesis and alizarin red S (Sigma Aldrich, St. Louis, MO, USA) staining for calcium ion visualization in osteogenesis were performed.

### 4.4. Surgical Procedure and Development of Biliary Obstruction

A model for the development of biliary obstruction was prepared and performed in accordance with the laws of the Czech Republic, which are comparable with the legislature of the European Union. The number of approval was MŠMT 23097/2013-4. The experimental animal used was *Sus Scrofa*, about 2 months of age and approximately 20 kg. The pigs were acclimatized before each experiment.

#### 4.4.1. Animal Preparation

Pigs (MSC group = 10 pigs; CONTROL group = 11 pigs) were starved for 12 h before surgical procedure and premedicated intramuscularly with 1.5 mg of atropine and 1.0 mg/kg of azaperone. Anesthesia was administered continuously through a peripheral venous catheter in the following average doses: 1.0 mg/kg/h of azaperone, 10 mg/kg/h of thiopental, 5–10 mg/kg/h of ketamine and 1–2 ug/kg/h of fentanyl. Muscle relaxation was provided by a bolus of 0.1–0.2 mg/kg of pancuronium at the beginning of surgery. Pigs were intubated and mechanically ventilated during surgical procedure, and electrocardiogram and oxygen saturation were monitored. The surgical procedure was performed under aseptic and antiseptic conditions. A total of 1.2 g of amoxicillin and clavulanic acid was administered as antibiotic prophylaxis in two doses (before surgery and 2 h after surgery). The abdomen was disinfected by an iodine solution, and the entire procedure was performed under sterile conditions.

#### 4.4.2. Surgical Procedure

The surgical procedure began with collection of bone marrow in a 1:1 solution of saline and heparin from the tuberositas tibiae, which was used for MSC isolation, followed by middle laparotomy. The skin was disrupted by a scalpel and the muscle and fascia were disrupted by electrocautery. A classical retrograde cholecystectomy was performed, and the arteria hepatica and ductus choledochus were disrupted. The ductus choledochus was centrally ligated with two ligatures and fixed with Fogarty’s catheter number 6 using four plastic clips. The Fogarty’s catheter balloon was filled with 2 mL of saline. The proximal end of Fogarty’s catheter was subcutaneously passed on the right side of the pig and introduced into the implantofix. The balloon was adjusted and inflated. The laparotomy and the incision on the right side were then closed in the anatomical layers.

#### 4.4.3. Postoperative Treatment

After the operation, the pigs were moved to a heated hutch and provided with ad libitum access to water and food. The pigs received a crushed pill of 20 mg pantoprazole each day to prevent gastroduodenal ulcus. The balloon initially remained filled for 13 days. On Day 13, the balloon was deflated under intramuscular anesthesia using 3–3.5 mL of Stresnil and 3–3.5 mL of Calypsol. Blood samples were measured for clinical and biochemical parameters (aspartate aminotransferase (AST), alanine aminotransferase (ALT), γ-glutamyl transferase (GGT), alkaline phosphatase (ALP), bilirubin, urea, creatinine, albumin and ammonia). The states of the bile duct and balloon were confirmed by ultrasonography (ultrasound machine Medison Sonoace 9900, convex probe with frequency 3.5 MHz). After Day 13, the Fogarty’s catheter balloon was alternately filled and emptied with saline each week for 9 weeks.

#### 4.4.4. Liver Resection and MSC Transplantation

After 9 weeks repeatedly filling and emptying the balloon, the pigs underwent partial liver resection and MSC transplantation. General anesthesia for the procedure was used as described above. At the beginning of the procedure, an implantofix was introduced into the internal or external jugular vein and used for general anesthesia during liver resection and postoperative care and observation. Middle laparotomy was performed in the original scar after excision. The position of Fogarty’s catheter was checked, and the catheter was removed after relieving the obstruction. Liver resection was performed by removing the left lobe using the Pringle maneuver followed by reconstruction of the bile ducts by Roux-en-Y choledochojejunostomy. Then, 1.2 g of amoxicillin and clavulanic acid were administered as antibiotic prophylaxis divided into two doses (before surgery and 2 h after surgery). Blood samples were collected before the operation, during liver resection and 2 h after resection. After resection, the MSC group of pigs (*n* = 10) received a suspension of allogenic MSC (10^6^ cells/kg in 100 mL of physiological solution) through the vena portae, while the control group (*n* = 11) received only 100 mL of physiological solution. A polypropylene mesh was used to close the laparotomy incision in the anatomical layer. Ultrasonographic controls were measured immediately after operation, and liver biopsy samples were taken for histological measurement.

#### 4.4.5. Postoperative Observation

Experimental pigs recovered in a warm hutch. Experimental and control groups were kept under the same conditions with ad libitum access to food and water. Blood samples were collected on the 1st, 3rd, 7th, 10th and 14th day after resection under anesthesia via implantofix. Ultrasonographic controls of liver parenchyma and the bile duct were conducted at the same time points. The experiment was terminated on the 14th day after liver resection by sacrificing the pigs under general anesthesia with a concentrated solution of potassium chloride, administered via the central venous catheter. Samples of liver parenchyma were taken for histological examination. 

### 4.5. Volumetry

Ultrasonographic examinations were performed at 0 h, 3 days, 7 days, 10 days and 14 days after hepatectomy. The diameters of the hypertrophic lobes were measured in B-mode in all three basic planes (axial, sagittal and coronal). The volume of the lobes was assessed by using the standard ultrasonographic formula used in human medicine (axial × sagittal × coronal/2).

### 4.6. Biochemical Analysis of Liver Enzymes

Blood samples were collected at 0 h, 3 days, 7 days, 10 days and 14 days after resection. Biochemical serum parameters were assessed focusing on liver function to detect the influence of the applied monoclonal antibody on the animals and to recognize possible differences between the experimental and control groups. Serum levels of albumin, bilirubin, urea, creatinine, AST, ALT, ALP and GGT were assessed by an Olympus 2700 biochemical analyzer (Olympus, Tokyo, Japan).

### 4.7. Quantification of Cytokines and Growth Factors in Plasma

Peripheral blood samples were collected at 0 h, 2 h, 1 day, 3 days and 14 days after resection and concentrations of IL 6, IL 8, TNF-α and TGF-β were determined by ELISA (e-Bioscience, Thermo Fisher Scientific, Waltham, MA, USA) or Luminex assay (e-Bioscience, Thermo Fisher Scientific, Waltham, MA, USA), according to manufacturer instructions.

### 4.8. Sampling and Histological Staining of Liver Tissue

For histological analysis, ten tissue block samples were collected from the livers of pigs who received MSC therapy, and eleven samples were collected from control pigs. All samples were taken at two time points, at the beginning of the experiment (resected liver on Day 0) and at the end (regenerated liver on Day 14). In total, 42 tissue blocks were collected and fixed in 10% phosphate-buffered formalin. The histological cutting plane of each sample was randomized using the orientator [44,45]. The samples were then dehydrated, embedded onto paraffin blocks and cut into 5 µm-thick histological sections. Overall morphology was assessed using hematoxylin–eosin staining. Connective tissue was contrasted with aniline blue and nuclear fast red staining. The outlines of individual hepatocytes were stained using a combination of alcian blue and Periodic Acid Schiff (PAS) reaction. For quantitative analysis, 398 sections were used in total. From each section stained with alcian blue, one field of view with a random position was recorded using the PlanC N 4×/0.1 microscope objective in order to quantify the volume fractions of connective tissue and area of the lobules (Figure 6A). In sections stained with PAS, systematic random uniform sampling [46,47,48] of each fifteenth field of view was done using the PlanC N 40×/0.65 objective. This sampling resulted in 2113 micrographs used to determine the morphometry of hepatocytes. From each micrograph, hepatocytes were sampled using four unbiased counting frames (Figure 6B).

### 4.9. Quantitative Morphometric Analysis of Liver Tissue

Histological parameters listed in Table 2 were used for a quantitative analysis. The A(lobule), V(MH) and V(PH) [49] were estimated using the nucleator stereological probe on two-dimensional images [50]. Briefly, this method approximates the area or volume of spatial structures with geometric circles and spheres, thereby estimating their cross-sectional areas with multiple measurements of their radiuses (Figure 6A,B). The measurement was done within the central regions of cells with clearly visible nuclei and nucleoli. Only cells selected by the unbiased counting frames were measured. From the measurements of these cross-sectional areas, the volume of hepatocytes was estimated. The volume fractions V_V_ (connective tissue, liver), V_V_ (hepatocytes, liver), V_V_ (MH, liver) and V_V_ (PH, liver) were determined using the point grid method [51,52]. All quantitative analyses were done using Ellipse stereological software (ViDiTo, Kosice, Slovakia).

### 4.10. Statistical Analysis

The MSC and control groups contained 10 and 11 individual pigs, respectively. All experimental data are expressed as mean ± standard deviation. The Shapiro–Wilk test was used to ascertain data normality. The statistical significance of differences between the MSC and control groups was determined by the Wilcoxon matched pairs test (*p* < 0.05) for histological analysis and by the Mann–Whitney U test of cross-ranked values (*p* < 0.05) for the remaining analyses. All statistical analyses were performed in Statistica v12 software (Tibco Software, Palo Alto, CA, USA).

## 5. Conclusions

Our study established the first large animal (porcine) model of repeated biliary obstruction by using Fogarty’s catheter to generate cyclic obstruction. In this model, allogenic MSC transplantation via the portal vein improved liver regeneration by means of liver volume growth, accompanied by increased mononuclear hepatocyte content in regenerated liver tissue. Liver function, analyzed by serum levels of liver enzymes (AST, ALT, ALP and GGT) and liver metabolites (albumin, bilirubin, urea and creatinine), was not affected by MSC transplantation. Even plasma pro-inflammatory cytokines (IL-6, IL-8 and TNF-α) and TGF-β remained unchanged upon MSC transplantation. This study underscores the impact of diverse experimental setups, especially large animal models, prior to clinical trials, as well as the need for careful evaluation of MSC application in liver disease therapy.

## Figures and Tables

**Table 1:** Correlations between histological morphometric parameters in the MSC group and in the control group. Spearman coefficients of significant correlations (*p* < 0.05) are presented.

Parameter 1	Parameter 2	WITH MSC		CONTROL	
		RESECTED	REGENERATED	RESECTED	REGENERATED
*V_V_ (hepatocytes,liver)*	*V_V_ (BB,liver)*	−0.76	−0.71	−0.82	−0.93
	*V_V_ (MH,liver)*	-	0.69	0.66	0.23
	*V_V_ (PH,liver)*	-	−0.69	−0.66	−0.23
*V (MH)*	*V (PH)*	0.65	0.72	0.78	0.61
*V_V_ (MH,liver)*	*V_V_ (PH,liver)*	−0.96	-	-	-
*V_V_ (connective,liver)*	*V (MH)*	-	−0.78	-	-
	*V (PH)*	-	−0.70	-	-

**Table 2:** Stereological principles, histological staining and sampling methods of photographs for quantitative parameters of liver morphometry. To maximize the reference space for each parameter, the lowest possible magnification was used that provided a resolution which guaranteed reliable visual control of the structures under study.

Abbreviation	Parameter (Unit)	Stereological Principle Used for Quantification	Section Staining and Objective Magnification
*A(lobule)*	Mean cross-sectional area of classical morphological lobules (mm^2^)	Step 1. Systematic uniform random sampling of one lobule per tissue section for quantification. Step 2. Nucleator probe in isotropic uniform random (IUR) sections.	Anilin blue and nuclear red objective 2×
*V_V_(connective, liver)*	Volume fraction of connective tissue in the liver (%)	Step 1. Systematic uniform random sampling of microscopic image fields selected for quantification from multiple physical sections. Step 2. Point grid and Cavalieri of Delesse principle.	Anilin blue and nuclear red objective 2×
*V_V_(hepatocytes, liver)*	Volume fraction of hepatocytes in the liver (%)	Step 1. Systematic uniform random sampling of microscopic image fields selected for quantification from multiple physical sections. Step 2. Point grid and Cavalieri of Delesse principle.	PAS objective 40×
*V_V_(MH,liver)*	Volume fraction of mononuclear hepatocytes in the liver (%)	Step 1. Systematic uniform random sampling of microscopic image fields selected for quantification from multiple physical sections. Step 2. Point grid and Cavalieri of Delesse principle.	PAS objective 40×
*V_V_(PH,liver)*	Volume fraction of polynuclear hepatocytes in the liver (%)	Step 1. Systematic uniform random sampling of microscopic image fields selected for quantification from multiple physical sections. Step 2. Point grid and Cavalieri of Delesse principle.	PAS objective 40×
*V_V_(BB,liver)*	Volume fraction of blood and biliary vessels (%)	The parameter was calculated by subtracting the total liver volume and the fractions of connective tissue and hepatocytes from 1.	-
*V(MH)*	Mean volume of mononuclear hepatocytes (µm^3^)	Step 1. Systematic uniform random sampling of the lobules (at least 30 per tissue section) selected for quantification. Step 2. Nucleator probe in isotropic uniform random (IUR) sections.	PAS objective 40×
*V(PH)*	Mean volume of polynuclear hepatocytes (µm^3^)	Step 1. Systematic uniform random sampling of the lobules (at least 30 per tissue section) selected for quantification. Step 2. Nucleator probe in isotropic uniform random (IUR) sections.	PAS objective 40×

## References

[1] Johnson S.R., Koehler A., Pennington L.K., Hanto D.W. (2000). Long-term results of surgical repair of bile duct injuries following laparoscopic cholecystectomy. Surgery.

[2] Ardiles V., McCormack L., Quinonez E., Goldaracena N., Mattera J., Pekolj J., Ciardullo M., de Santibanes E. (2011). Experience using liver transplantation for the treatment of severe bile duct injuries over 20 years in Argentina: Results from a National Survey. HPB.

[3] Barbier L., Souche R., Slim K., Ah-Soune P. (2014). Long-term consequences of bile duct injury after cholecystectomy. J. Visc. Surg..

[4] De Santibanes E., Ardiles V., Gadano A., Palavecino M., Pekolj J., Ciardullo M. (2008). Liver transplantation: The last measure in the treatment of bile duct injuries. World J. Surg..

[5] Lubikowski J., Chmurowicz T., Post M., Jarosz K., Bialek A., Milkiewicz P., Wojcicki M. (2012). Liver transplantation as an ultimate step in the management of iatrogenic bile duct injury complicated by secondary biliary cirrhosis. Ann. Transpl..

[6] Ruemmele P., Hofstaedter F., Gelbmann C.M. (2009). Secondary sclerosing cholangitis. Nat. Rev. Gastroenterol. Hepatol..

[7] Negi S.S., Sakhuja P., Malhotra V., Chaudhary A. (2004). Factors predicting advanced hepatic fibrosis in patients with postcholecystectomy bile duct strictures. Arch. Surg..

[8] Lazaridis K.N., Gores G.J., Lindor K.D. (2001). Ursodeoxycholic acid “mechanisms of action and clinical use in hepatobiliary disorders”. J. Hepatol..

[9] Nordin A., Halme L., Makisalo H., Isoniemi H., Hockerstedt K. (2002). Management and outcome of major bile duct injuries after laparoscopic cholecystectomy: From therapeutic endoscopy to liver transplantation. Liver Transpl..

[10] Patkowski W., Skalski M., Zieniewicz K., Nyckowski P., Smoter P., Krawczyk M. (2010). Orthotopic liver transplantation for cholestatic diseases. Hepatogastroenterology.

[11] Pottakkat B., Vijayahari R., Prakash A., Singh R.K., Behari A., Kumar A., Kapoor V.K., Saxena R. (2010). Factors predicting failure following high bilio-enteric anastomosis for post-cholecystectomy benign biliary strictures. J. Gastrointest Surg..

[12] Loinaz C., Gonzalez E.M., Jimenez C., Garcia I., Gomez R., Gonzalez-Pinto I., Colina F., Gimeno A. (2001). Long-term biliary complications after liver surgery leading to liver transplantation. World J. Surg..

[13] Schwartz S.I. (1981). Biliary tract surgery and cirrhosis: A critical combination. Surgery.

[14] Chapman W.C., Halevy A., Blumgart L.H., Benjamin I.S. (1995). Postcholecystectomy bile duct strictures. Management and outcome in 130 patients. Arch. Surg..

[15] Fang B., Shi M., Liao L., Yang S., Liu Y., Zhao R.C. (2004). Systemic infusion of FLK1(+) mesenchymal stem cells ameliorate carbon tetrachloride-induced liver fibrosis in mice. Transplantation.

[16] Li T., Zhu J., Ma K., Liu N., Feng K., Li X., Wang S., Bie P. (2013). Autologous bone marrow-derived mesenchymal stem cell transplantation promotes liver regeneration after portal vein embolization in cirrhotic rats. J. Surg. Res..

[17] Higashiyama R., Inagaki Y., Hong Y.Y., Kushida M., Nakao S., Niioka M., Watanabe T., Okano H., Matsuzaki Y., Shiota G. (2007). Bone marrow-derived cells express matrix metalloproteinases and contribute to regression of liver fibrosis in mice. Hepatology.

[18] Oyagi S., Hirose M., Kojima M., Okuyama M., Kawase M., Nakamura T., Ohgushi H., Yagi K. (2006). Therapeutic effect of transplanting HGF-treated bone marrow mesenchymal cells into CCl4-injured rats. J. Hepatol..

[19] Amin M.A., Sabry D., Rashed L.A., Aref W.M., el-Ghobary M.A., Farhan M.S., Fouad H.A., Youssef Y.A. (2013). Short-term evaluation of autologous transplantation of bone marrow-derived mesenchymal stem cells in patients with cirrhosis: Egyptian study. Clin. Transpl..

[20] Jang Y.O., Kim Y.J., Baik S.K., Kim M.Y., Eom Y.W., Cho M.Y., Park H.J., Park S.Y., Kim B.R., Kim J.W. (2014). Histological improvement following administration of autologous bone marrow-derived mesenchymal stem cells for alcoholic cirrhosis: A pilot study. Liver Int..

[21] Gerling B., Becker M., Waldschmidt J., Rehmann M., Schuppan D. (1996). Elevated serum aminoterminal procollagen type-III-peptide parallels collagen accumulation in rats with secondary biliary fibrosis. J. Hepatol..

[22] Heller J., Trebicka J., Shiozawa T., Schepke M., Neef M., Hennenberg M., Sauerbruch T. (2005). Vascular, hemodynamic and renal effects of low-dose losartan in rats with secondary biliary cirrhosis. Liver Int..

[23] Raetsch C., Jia J.D., Boigk G., Bauer M., Hahn E.G., Riecken E.O., Schuppan D. (2002). Pentoxifylline downregulates profibrogenic cytokines and procollagen I expression in rat secondary biliary fibrosis. Gut.

[24] Chen C.Y., Shiesh S.C., Wu M.C., Lin X.Z. (1999). The effects of bile duct obstruction on the biliary secretion of ciprofloxacin in piglets. Am. J. Gastroenterol..

[25] Daneze E.R., Terra G.A., Terra J.A., Campos A.G., Silva A.A., Terra S.A. (2011). Comparative study between ligature with thread or metallic clamping by means of laparoscopy with the purpose of experimental biliary obstruction in swines. Acta Cir. Bras..

[26] Shamiyeh A., Vattay P., Tulipan L., Schrenk P., Bogner S., Danis J., Wayand W. (2004). Closure of the cystic duct during laparoscopic cholecystectomy with a new feedback-controlled bipolar sealing system in case of biliary obstruction—An experimental study in pigs. Hepatogastroenterology.

[27] Adas G., Koc B., Adas M., Duruksu G., Subasi C., Kemik O., Kemik A., Sakiz D., Kalayci M., Purisa S. (2016). Effects of mesenchymal stem cells and VEGF on liver regeneration following major resection. Langenbecks Arch. Surg..

[28] Mohamadnejad M., Alimoghaddam K., Mohyeddin-Bonab M., Bagheri M., Bashtar M., Ghanaati H., Baharvand H., Ghavamzadeh A., Malekzadeh R. (2007). Phase 1 trial of autologous bone marrow mesenchymal stem cell transplantation in patients with decompensated liver cirrhosis. Arch. Iran Med..

[29] Carvalho A.B., Quintanilha L.F., Dias J.V., Paredes B.D., Mannheimer E.G., Carvalho F.G., Asensi K.D., Gutfilen B., Fonseca L.M., Resende C.M. (2008). Bone marrow multipotent mesenchymal stromal cells do not reduce fibrosis or improve function in a rat model of severe chronic liver injury. Stem. Cells.

[30] Suk K.T., Yoon J.H., Kim M.Y., Kim C.W., Kim J.K., Park H., Hwang S.G., Kim D.J., Lee B.S., Lee S.H. (2016). Transplantation with autologous bone marrow-derived mesenchymal stem cells for alcoholic cirrhosis: Phase 2 trial. Hepatology.

[31] Verstegen M.M.A., Mezzanotte L., Yanto Ridwan R., Wang K., De Haan J., Schurink I.J., Sierra Parraga J.M., Hoogduijn M., Kessler B.M., Huang H. (2020). First report on ex vivo delivery of paracrine active human mesenchymal stromal cells to liver grafts during machine perfusion. Transplantation.

[32] De Witte S.F.H., Luk F., Sierra Parraga J.M., Gargesha M., Merino A., Korevaar S.S., Shankar A.S., O’Flynn L., Elliman S.J., Roy D. (2018). Immunomodulation by Therapeutic Mesenchymal Stromal Cells (MSC) Is Triggered Through Phagocytosis of MSC By Monocytic Cells. Stem. Cells.

[33] You Y., Zhang J., Gong J., Chen Y., Li Y., Yang K., Liu Z. (2015). Mesenchymal stromal cell-dependent reprogramming of Kupffer cells is mediated by TNF-alpha and PGE2 and is crucial for liver transplant tolerance. Immunol. Res..

[34] Huang B., Cheng X., Wang H., Huang W., la Ga Hu Z., Wang D., Zhang K., Zhang H., Xue Z., Da Y. (2016). Mesenchymal stem cells and their secreted molecules predominantly ameliorate fulminant hepatic failure and chronic liver fibrosis in mice respectively. J. Transl. Med..

[35] Jang Y.O., Kim M.Y., Cho M.Y., Baik S.K., Cho Y.Z., Kwon S.O. (2014). Effect of bone marrow-derived mesenchymal stem cells on hepatic fibrosis in a thioacetamide-induced cirrhotic rat model. BMC Gastroenterol..

[36] Forbes S.J., Russo F.P., Rey V., Burra P., Rugge M., Wright N.A., Alison M.R. (2004). A significant proportion of myofibroblasts are of bone marrow origin in human liver fibrosis. Gastroenterology.

[37] Russo F.P., Alison M.R., Bigger B.W., Amofah E., Florou A., Amin F., Bou-Gharios G., Jeffery R., Iredale J.P., Forbes S.J. (2006). The bone marrow functionally contributes to liver fibrosis. Gastroenterology.

[38] Junatas K.L., Tonar Z., Kubikova T., Liska V., Palek R., Mik P., Kralickova M., Witter K. (2017). Stereological analysis of size and density of hepatocytes in the porcine liver. J. Anat..

[39] Cupertino M.C., Costa K.L., Santos D.C., Novaes R.D., Condessa S.S., Neves A.C., Oliveira J.A., Matta S.L. (2013). Long-lasting morphofunctional remodelling of liver parenchyma and stroma after a single exposure to low and moderate doses of cadmium in rats. Int. J. Exp. Pathol..

[40] De Freitas K.M., Almeida J.M., Monteiro J.C., Diamante M.A., Vale J.S., Camargo C., Jorge M.H., Dolder H. (2015). The effects of cyclosporin A and Heteropterys tomentosa on the rat liver. An. Acad. Bras. Cienc..

[41] Gorla G.R., Malhi H., Gupta S. (2001). Polyploidy associated with oxidative injury attenuates proliferative potential of cells. J. Cell Sci..

[42] Dai L.J., Li H.Y., Guan L.X., Ritchie G., Zhou J.X. (2009). The therapeutic potential of bone marrow-derived mesenchymal stem cells on hepatic cirrhosis. Stem Cell Res..

[43] Volarevic V., Nurkovic J., Arsenijevic N., Stojkovic M. (2014). Concise review: Therapeutic potential of mesenchymal stem cells for the treatment of acute liver failure and cirrhosis. Stem Cells.

[44] Mattfeldt T., Mall G., Gharehbaghi H., Moller P. (1990). Estimation of surface area and length with the orientator. J. Microsc..

[45] Nyengaard J.R., Gundersen H.J.G. (1992). The Isector—A Simple and Direct Method for Generating Isotropic, Uniform Random Sections from Small Specimens. J. Microsc..

[46] Marcos R., Monteiro R.A., Rocha E. (2012). The use of design-based stereology to evaluate volumes and numbers in the liver: A review with practical guidelines. J. Anat..

[47] Mouton P.R. (2011). Unbiased Stereology: A Concise Guide.

[48] Nyengaard J.R., Gundersen H.J.G. (2006). Sampling for stereology in lungs. Eur. Respir. Rev..

[49] Bruha J., Vycital O., Tonar Z., Mirka H., Haidingerova L., Benes J., Palek R., Skala M., Treska V., Liska V. (2015). Monoclonal antibody against transforming growth factor Beta 1 does not influence liver regeneration after resection in large animal experiments. In Vivo.

[50] Gundersen H.J., Jensen E.B. (1985). Stereological estimation of the volume-weighted mean volume of arbitrary particles observed on random sections. J. Microsc..

[51] Mik P., Tonar Z., Malečková A., Eberlová L., Liška V., Pálek R., Rosendorf J., Jiřík M., Mírka H., Králíčková M. (2018). Distribution of Connective Tissue in the Male and Female Porcine Liver: Histological Mapping and Recommendations for Sampling. J. Comp. Pathol..

[52] Palek R., Rosendorf J., Maleckova A., Vistejnova L., Bajcurova K., Mirka H., Tegl V., Brzon O., Kumar A., Bednar L. (2020). Influence of Mesenchymal Stem Cell Administration on the Outcome of Partial Liver Resection in a Porcine Model of Sinusoidal Obstruction Syndrome. Anticancer Res..

